# Metabolomics in the diagnosis of sepsis

**DOI:** 10.1186/1824-7288-40-S1-A11

**Published:** 2014-08-11

**Authors:** Vassilios Fanos, Mauro Stronati, Diego Gazzolo, Giovanni Corsello

**Affiliations:** 1 Neonatal Intensive Care Unit, Puericulture Institute and Neonatal Section, Azienda Ospedaliera Universitaria, University of Cagliari, Cagliari 09131, Italy; 2 Neonatal Unit and Neonatal Intensive Care Unit, Maternal-Infant Department, Fondazione IRCCS Policlinico San Matteo, Pavia, Italy; 3 Department of Maternal, Fetal and Neonatal Health, C. Arrigo Children's Hospital, Spalto Marengo 46, I- 15100 Alessandria, Italy; 4 Operative Unit of Pediatrics and Neonatal Intensive Therapy, Mother and Child Department, University of Palermo, Palermo, Italy

## Introduction

Sepsis is an important cause of mortality and morbidity for preterm and hospitalized newborn babies. Today, no single test satisfies the criteria as being the ideal marker for the early diagnosis of neonatal sepsis. Analysis of the entire metabolome is a promising method for determining metabolic variations correlated with sepsis [1-6].

## Metabolomics profiling and sepsis

Works on metabolomics concerning sepsis conducted on animals and humans of different ages (newborn and adults) have recently been published and are presented in Table 1. In septic patients compared to controls (in plasma and urine) it is possible to observe an increase of metabolites which are part of the oxidative metabolism of fatty acids (such as hydroxybutyrate, acylcarnitines and acetoacetate). Briefly stated, alterations in the glucose metabolism in critical conditions can be seen as a redistribution of glucose consumption from the mitochondrial oxidative phosphorylation to other metabolic pathways, such as the production of lactate and the pentose phosphate pathway. In the study by Fanos et al. [7] a combined approach based on both nuclear magnetic resonance (1)H-NMR) and gas-chromatography-mass spectrometry (GC-MS) techniques was used applied to neonatal infections. The study population included 25 neonates: 9 patients had a diagnosis of sepsis and 16 were healthy controls. This study showed a unique metabolic profile of the patients affected by sepsis compared to non-affected ones with a statistically significant difference between the two groups (p = 0.05). Mickiewicz et al [8] examined serum samples collected from 60 patients with septic shock (by Gram- and/or Gram+), 40 patients with SIRS and 40 healthy children by nuclear magnetic resonance spectroscopy spectra. Some of the metabolite concentrations were able to separate between patient groups. The main messages from the published studies are as follows. a) Metabolomics is able to early diagnose the infection (in some cases in preclinical conditions). b) Metabolomics is able to predict the outcome in single individuals and the AUC values are close to 1. c) Metabolomics appears to be a promising and useful instrument also in the diagnosis of sepsis. d) In the next future some easy tools, like urinary dipsticks, with the discriminant metabolites will be available in clinical settings, bedside.

**Table 1:** Metabolomic studies that have analyzed the metabolic profiles of septic patients and of experimental animals (From ref. 6, mod.)

Author	Population study	Sample	Metabolomic analysis	Metabolite alterations
**Fanos et al. 2014**	9 septic newborns vs 16 control newborns	Urine	GC-MS 1H NMR	Lactate, glucose, maltose, ribitol, ribonic acid, pseudo-uridine, 2,3,4 trihydroxybutiric acid, 2-ketpgluconic acid, 3,4 hydroxybutanoic acid, 3,4,5 trihydroxypentanoic acid <(GC-MS) Acetate, acetone, citrate, creatinine, glycine, lactate, lysine, glucose (1H-NMR)

**Mickiewicz et al. 2013**	60 septic shock vs 40 SIRS vs 40 control pediatric patients	Serum	1H-NMR	2-hydroxybutyrate, 2-hydroxyisovalerate, lactate, glucose, 2-oxoisocaproate, creatine, creatinine, histidine, and phenylalanine

**Schmerler et al. 2012**	74 SIRS vs 69 septic vs 16 control human adults	Blood	LC-MS/MS	Acylcarnitines and glycerophosphatidylcholines

**Mickiewitz et al. 2014**	39 septic shock adult patients vs 20 ICU control patients	Serum	1H-NMR	Isobutyrate, phenylalanine, 2 hydroxyisovalerate, myoinositol, acetylcarnitine, creatine, lactate, valine, arginine, methanol, glucose, glycine

**Liu et al. 2010**	40 septic vs control rats	Plasma	UPLC–Q-TOF-MS	Hypoxanthine, indoxyl sulfate, glucuronic acid, gluconic acid, proline, uracil, nitrotyrosine, uric acid and trihydroxy cholanoic acid

**Lin et al. 2009**	40 septic vs 20 control rats	Serum	1H NMR	Lactate, alanine, acetate, acetoacetate, hydroxybutyrate and formate

**Izquierdo-Garcìa et al. 2011**	14 septic vs 14 control rats	Lung tissue, BALF and serum	1H NMR	Alanine, creatine, phosphoethanolamine and myoinositol

## Conclusions

Present-day methods and procedures for the diagnosis of systemic neonatal infections are hindered by low sensitivity and long response times. Metabolomics is showing promise of becoming a most effective method, even in neonatology and paediatrics.

## References

[B1] Fanos V Van den Anker J Noto A Mussap M Atzori L Metabolomics in neonatology: fact or fiction? Semin Fetal Neonatal Med 2013 18 3 12 10.1016/j.siny.2012.10.014 23195852

[B2] Fanos V Antonucci R Atzori L Metabolomics in the developing infant Curr Opin Pediatr 2013 25 604 12 10.1097/MOP.0b013e328363ec8b 23995425

[B3] Buonocore G Mussap M Fanos V Proteomics and metabolomics: can they solve some mysteries of the newborn? J Matern Fetal Neonatal Med 2013 26 Suppl 2 7 8 24059543 10.3109/14767058.2013.832579

[B4] Mussap M Antonucci R Noto A Fanos V The role of metabolomics in neonatal and pediatric laboratory medicine Clin Chim Acta 2013 426 127 38 24035970 10.1016/j.cca.2013.08.020

[B5] Noto A Mussap M Fanos V Is _1_H NMR metabolomics becoming the promising early biomarker for neonatal sepsis and for monitoring the antibiotic toxicity J Chemother 2013 26 130 2 24112754 10.1179/1973947813Y.0000000149

[B6] Dessì A Corsello G Stronati M Gazzolo D Caboni P Carboni R Fanos V New diagnostic possibilities in systemic neonatal infections: metabolomics Early Hum Dev 2014 90 S1 S19 S2 24709449 10.1016/S0378-3782(14)70007-6

[B7] Fanos V Caboni P Corsello G Stronati M Gazzolo D Noto A Lussu M Dessì A Giuffrè M Lacerenza S Serraino F Garofoli F Serpero LD Liori B Carboni R Atzori L Urinary (1)H-NMR and GC-MS metabolomics predicts early and late onset neonatal sepsis Early Hum Dev 2014 90 S1 S78 83 24709468 10.1016/S0378-3782(14)70024-6

[B8] Mickiewicz B Vogel HJ Wong HR Winston BW Metabolomics as a novel approach for early diagnosis of pediatric septic shock and its mortality Am J Respir Crit Care Med 2013 187 967 76 10.1164/rccm.201209-1726OC 23471468 PMC3707368

